# Medical staff evaluation on ‘the effect of medical alliance policy on hierarchical diagnosis and treatment’

**DOI:** 10.3389/fpubh.2024.1366100

**Published:** 2024-05-15

**Authors:** Zijun Zhao, Xianyu Xie, Qinde Wu

**Affiliations:** ^1^School of Public Administration & Law, Fujian Agriculture and Forestry University, Fuzhou, Fujian, China; ^2^Department of Medical Administration, Fujian Medical University Union Hospital, Fuzhou, Fujian, China; ^3^Department of Education Administration, Fujian Medical University Union Hospital, Fuzhou, Fujian, China

**Keywords:** medical staff, medical alliance, hierarchical diagnosis and treatment, effect evaluation, medical reform

## Abstract

**Introduction:**

Medical alliances are essential for constructing an hierarchical diagnosis and treatment (HDT) system; therefore, it is crucial to promote such alliances and evaluate their effectiveness in this regard from the medical staff perspective. This study thus investigated and analyzed the evaluations of medical staff in China concerning the effect of medical alliances on promoting HDT with the intention to encourage further establishment of medical alliances and HDT under China’s new medical reform.

**Methods:**

A total of 616 medical staff personnel from 3 medical alliances in Fujian Province were surveyed, and data were analyzed using SPSS 20.0 software.

**Results:**

The level of medical institutions, posts and satisfaction with their medical alliances influenced the evaluation of medical alliance effectiveness in resolving the problem of expensive medical services. Primary medical institutions are more inclined toward policy formulation and related work; thus, the interests of primary hospitals can be guaranteed. However, tertiary hospitals must provide additional workforce, material, and financial resources to support primary hospitals.

**Discussion:**

Therefore, it is necessary to coordinate the interests of the medical staff at different levels of medical institutions. The study makes a significant contribution to the literature because it highlights the effect of medical alliances in promoting hierarchical diagnosis and treatment.

## 1 Introduction

In 2009, the Central Committee of the Communist Party of China and the State Council issued a new medical reform policy (1). The goal was to effectively alleviate the problem of “high medical service expenses for the masses” and gradually establish a system of hierarchical diagnosis and treatment (HDT) and two-way referrals. In 2015, the General Office of the State Council published the Guiding Opinions on Promoting the Development of the HDT System and specified the development objectives of the HDT. The construction of a reasonable and orderly HDT system is currently touted as an effective method of solving China’s high medical service expenses problem. Medical alliances are an essential measure for constructing an HDT system. Medical alliances are regional partnerships or mutually beneficial relationships led by general tertiary hospitals. They comprise several tertiary, secondary, and community health service centers (2), which help increase access to additional resources, achieve long-term sustainable savings, and expand reach in communities. Based on the principle of government-led overall planning, a consortium is formed according to the functions, positioning, and levels of different medical institutions. Medical alliances are necessary to promote people to realize the hierarchical diagnosis and treatment pattern of primary diagnosis, two-way referral, acute and chronic division, and upper and lower linkage in the process of medical treatment. It is imperative to focus on the effect of medical alliances on promoting the establishment of an HDT system and whether they have effectively solved the problem of high expenses of medical services for the masses.

Throughout the literature [i.e., (3–6)], many scholars have studied the operational mechanism of medical alliances from different angles. However, evaluation research on medical staff under the medical alliance mode is mostly sporadic, and no scholars have studied the incentive mechanism of medical staff from the perspective of medical alliances. Medical staff is the most flexible part of the medical alliance. How to effectively improve the satisfaction and work enthusiasm of medical staff has become a major issue for the stable development of medical alliances. Medical staff undertake various tasks, including creating schemes, coordinating and organizing referrals, and teaching and training fellows and residents as essential components of medical alliances (7). Therefore, it is crucial to promote and popularize the establishment of medical alliances and evaluate and analyze the effect of these medical alliances on establishing an HDT system from the medical staff perspective (8). This study evaluated the understanding of medical staff in Fujian Province regarding the effect of medical alliances on establishing an HDT system as part of medical reform. The objective was to provide a reference for the role of medical alliances in promoting HDTs in Fujian Province and provide a reference for relevant management personnel in formulating policies and further promoting the formation of medical alliances and HDTs.

## 2 Materials and methods

### 2.1 Source of materials

Participants were selected based on the following inclusion criteria: their medical institutions had established medical alliances for more than 5 years, aged 18 years or older, a minimum of 3 years of working experience, and were on duty during the survey. A stratified sampling method was used to select typical and representative medical institutions from nine cities in Fujian Province, including three provincial hospitals, five municipal hospitals, four county hospitals, and nine community medical service or township health centers (see below for explanation of medical institution levels). A total of 21 hospitals at all levels were used as sample institutions. The criteria of stratified sampling were applied according to the proportion of medical staff in each city and each member unit of the medical alliances. The proportion of personnel was randomly selected according to the probability-proportional-to-size sampling method, resulting in 321 people from tertiary hospitals (municipal hospital or provincial hospital), 175 people from secondary hospitals (county-level hospital), and 120 people from primary hospitals (community medical service center or township health center).

### 2.2 Medical institution levels

Community medical service centers or township health centers: These are primary health care institutions that directly provide comprehensive medical, prevention, rehabilitation, and health services for communities. The total number of these institutions’ hospital beds ranges from 20 to 99.

County-level hospitals: These are regional hospitals providing medical and health services to several communities and technical centers for regional medical prevention. The total number of their hospital beds ranges from 100 to 499.

Municipal and provincial hospitals: These provide medical and health services across regions, provinces, cities, and the entire country. They are medical prevention technology centers with comprehensive medical, teaching, and scientific research capabilities. The total number of their beds is more than 500. Municipal hospitals mainly serve patients in the city area, while provincial hospitals provide medical services for the entire province and in the surrounding provinces and cities.

### 2.3 Study methods

#### 2.3.1 Questionnaire survey method

Based on the social division of labor and stakeholder theory, self-compiled questionnaires were used to collect information and materials on the medical staff’s evaluation of medical alliances in promoting the HDT system. The questionnaire content included basic information, an evaluation of medical alliances in promoting HDT, and suggestions for improvement. The basic information included medical institution level, medical staff post and professional title, workload changes after medical alliance establishment, and satisfaction with medical alliances. The evaluation of medical alliances in promoting HDT included effect evaluation of medical alliances in promoting the establishment of HDT and solving the problem of high expenses for medical services. Suggestions for improvement included ideas for further measures to improve the formation of medical alliances.

#### 2.3.2 Statistical analysis method

Epidata 3.1 was used to input the questionnaire data and SPSS 20.0 was applied to analyze the data. Measurement data conforming to a normal distribution were expressed as the mean ± standard deviation and compared using an independent sample t-test. Measurement data that did not conform to a normal distribution were expressed as medians (lower quartiles and upper quartiles) and compared using the Kruskal–Wallis and Mann–Whitney *U* rank sum tests. Count data were expressed as rates and compared using the *χ*2 test. *p* < 0.05 was considered statistically significant. Univariate analysis and multivariate linear regression analysis were applied.

We analyzed the reliability of the questionnaire using Cronbach’s alpha coefficient and obtained a coefficient of 0.823; thus, the reliability level was high. The validity of the questionnaire was analyzed and measured using the KMO index. The KMO index value was 0.875, the Bartlett sphericity test chi-square value was 4138.527, and the *p*-value was 0.000. The validity level was considered good. The reliability and validity of the questionnaire passed the test and were suitable for questionnaire analysis.

#### 2.3.3 Quality control

The surveyors were extensively trained and confidentiality was confirmed before administration of the questionnaire. Each questionnaire was uniformly numbered and reviewed repeatedly and carefully. Therefore, unqualified questionnaires with incomplete information or contradictory answers were removed and excluded. Questionnaires were entered dually to ensure data entry quality.

## 3 Results

### 3.1 Basic information about the medical staff

A total of 700 questionnaires were distributed and 656 were collected with a recovery rate of 93.71%. Among them, 616 questionnaires were found to be valid, with an effective rate of 93.90%. Table 1 shows the details of the 616 respondents. Most respondents were employees of municipal hospitals (35.71%), followed by county-level hospitals (28.41%), community medical services or township health centers (19.48%), and provincial hospitals (16.40%). The posts held by the respondents were mainly clinical positions, accounting for 45.45%. The respondents mainly comprised medical staff personnel with junior professional titles (40.91%). Regarding changes in workload after the medical alliance establishment, 53.25% experienced no change, 43.83% experienced an increase, and only 2.92% experienced a decrease. In terms of satisfaction with the medical alliances, 55.19% were satisfied, 40.10% were not satisfied, and 4.71% were generally dissatisfied (Table 1).

**Table 1:** Basic information of medical staff surveyed.

Basic characteristics	*N*	%
Medical institutions level		
Community medical service center or township health center	120	19.48
County-level hospital	175	28.41
Municipal hospital	220	35.71
Provincial hospital	101	16.40
Posts		
Clinical	280	45.45
Medical technology	66	10.71
Nursing	183	29.71
Administration and logistics	87	14.12
Professional titles		
Senior	98	15.91
Intermediate	211	34.25
Junior	252	40.91
None	55	8.93
Workload changes after the medical alliances’ establishment		
No change	328	53.25
Increase	270	43.83
Decrease	18	2.92
Satisfaction with own medical alliances		
Satisfied	340	55.19
Not satisfied	247	40.10
General satisfied	29	4.71

### 3.2 Evaluation of medical alliances in promoting HDT

Regarding the effective evaluation of medical alliances in promoting the establishment of HDTs, among the 616 respondents, 17.37% (*n* = 107) considered it to have no effect, 50.00% (*n* = 308) assessed it as having an average effect, 25.65% (*n* = 158) considered it to have a good effect, and 6.98% (*n* = 43) considered the effect very good.

In terms of the effective evaluation of medical alliances in solving the problem of high expenses for medical services, of the 616 respondents, 37.50% (*n* = 231) considered the effect average, 28.73% (*n* = 177) considered the effect good, 16.07% (*n* = 99) considered the problem unsolvable, and 17.69% (*n* = 109) believed that the effect was uncertain.

### 3.3 Effect evaluation of medical alliances in promoting the establishment of HDT

Univariate analysis showed significant differences in the effect evaluation of medical alliances in promoting the establishment of HDT with different levels of satisfaction with one’s own medical alliances (*p* < 0.05). The differences in the effect evaluations of medical alliances in promoting the establishment of HDTs with different medical institution levels, posts, professional titles, and workload changes after medical alliance establishment were not significant (*p* > 0.05) (Table 2).

**Table 2:** Univariate analysis of the effect evaluation of medical alliances in promoting the establishment of HDTs (*n*/%).

Name	No effect	Initial effect	Good effect	Very good effect	*χ*2	*p*
Medical institution level					14.955	0.093
Community medical service center or township health center	14/11.67	56/46.67	39/32.5	11/9.17		
County-level hospital	31/17.71	91/52.00	45/25.71	8/4.57		
Municipal hospital	36/16.36	109/49.55	57/25.91	18/8.18		
Provincial hospital	26/25.74	52/51.49	17/16.83	6/5.94		
Posts					11.196	0.257
Clinical	60/21.43	131/46.79	72/25.71	17/6.07		
Medical technology	9/13.64	38/57.58	15/22.73	4/6.06		
Nursing	23/12.57	90/49.18	54/29.51	16/8.74		
Administration and logistics	15/17.24	49/56.32	17/19.54	6/6.90		
Professional titles						
Senior	26/26.53	42/42.86	24/24.49	6/6.12	13.515	0.129
Intermediate	33/15.64	116/54.98	48/22.75	14/6.64		
Junior	34/13.49	126/50.00	73/28.97	19/7.54		
No title	14/25.45 4/10.81	24/43.64	13/23.64	4/7.27		
Workload changes after the establishment of medical alliances					9.450	0.130
No change	58/17.68	170/51.83	73/22.26	27/8.23		
Increase	47/17.41	131/48.52	79/29.26	13/4.81		
Decrease	2/11.11	7/38.89	6/33.33	3/16.67		
Satisfaction with medical alliances					223.262	<0.01
Satisfied	14/4.12	153/45.00	133/39.12	40/11.76		
General satisfied	68/27.53	15/61.13	25/10.12	3/1.21		
Not satisfied	25/86.21	4/13.79	0/0.00	0/0.00		

### 3.4 Effect evaluation of medical alliances in solving the problem of high expenses for medical services

Univariate analysis showed that the differences in the effect evaluation of medical alliances in solving the problem of high expenses for medical services with different medical institution levels, staff posts, professional titles, and satisfaction with one’s own medical alliances and medical institutions were significant (*p* < 0.05). The differences in the effect evaluation of medical alliances in solving the problem of high expenses for medical services with different workload changes after medical alliance establishment were insignificant (*p* > 0.05) (Table 3).

**Table 3:** Univariate analysis of the effect evaluation of medical alliances in solving the problem of “high expenses for medical services” (*n*/%).

Name	Yes, but the effect is average	Yes, the effect is good	Uncertain	No	*χ*2	*p*
Medical institution level					28.800	0.001
Community medical service center or township health center	51/42.50	48/40.00	13/10.83	8/6.67		
County-level hospital	70/40.00	42/24.00	32/18.29	31/17.71		
Municipal hospital	81/36.82	64/29.09	40/18.18	35/15.91		
Provincial hospital	29/28.71	23/22.77	24/23.76	25/24.75		
Posts					25.971	0.002
Clinical	90/32.14	77/27.5	50/17.86	63/22.5		
Medical technology	28/42.42	21/31.82	9/13.64	8/12.12		
Nursing	71/38.80	61/33.33	29/15.85	22/12.02		
Administration and logistics	42/48.28	18/20.69	21/24.14	6/6.90		
Professional titles					17.744	0.035
Senior	42/42.86	16/16.33	16/16.33	24/24.49		
Intermediate	70/33.18	68/32.23	39/18.48	34/16.11		
Junior	98/38.89	80/31.75	40/15.87	34/13.49		
No title	21/38.18	13/23.64	14/25.45	7/12.73		
Workload changes after the establishment of medical alliances					9.438	0.141
No change	120/36.59	93/28.35	67/20.43	48/14.63		
Increase	106/39.26	74/27.41	41/15.19	49/18.15		
Decrease	5/27.78	10/55.56	1/5.56	2/11.11		
Satisfaction with own medical alliances						
Satisfied	128/37.65	164/48.24	35/10.29	13/3.82	238.766	<0.01
General satisfied	97/39.27	13/5.26	71/28.74	66/26.72		
Not satisfied	6/20.69	0/0.00	3/10.34	20/68.97		

According to univariate analysis, the differences in medical institution levels, staff posts, professional titles, and satisfaction with one’s own medical alliances were significant. Multiple regression analysis was performed considering effect evaluation scores as dependent variables and medical institution levels, staff posts, professional titles, and satisfaction with one’s own medical alliances as independent variables, as shown in Table 4.

**Table 4:** Variable assignment table.

Variable	Description of valuation
Effect evaluation of medical alliances in solving the problem of high expenses for medical services	Effect evaluation scores Yes, the effect is good = 4, Yes, but the effect is average = 3, Uncertain = 2, No = 1
Medical institution level	Community medical service center or township health center = 1, County-level hospital = 2, Municipal hospital = 3, Provincial hospital = 4
Posts	Clinical = 1, Medical technology = 2, Nursing = 3, Administration and logistics = 4
Professional titles	Senior = 1, Intermediate = 2, Junior = 3, No title = 4
Satisfaction with own medical alliances	Satisfied = 1, Generally satisfied = 2, Not satisfied = 3

This analysis revealed the statistical significance of the determination coefficient (*F* = 293.899, *p* < 0.001), showed that the model was statistically significant. Posts, Medical institution level and satisfaction with their medical alliances influenced the respondents’ evaluation of the effect of medical alliances in resolving the problem of high expenses for medical services (Table 5).

**Table 5:** Results of multiple regression analysis.

Refer = Effect is average	Factors	*B*	SE	Wald	*p*	OR	95%CI of OR	
							Lower	Upper
Effect is good	Posts (Refer = Administration and logistics)							
	Clinical	0.619	0.361	2.946	0.086	1.858	0.916	3.767
	Medical technology	0.685	0.449	2.324	0.127	1.984	0.822	4.787
	Nursing	0.611	0.372	2.696	0.101	1.842	0.888	3.819
	Professional titles (Refer = No title)							
	Senior	−0.835	0.518	2.598	0.107	0.434	0.157	1.198
	Intermediate	0.119	0.444	0.072	0.789	1.126	0.472	2.689
	Junior	−0.16	0.432	0.137	0.712	0.852	0.365	1.989
	Medical institution level (Refer = Provincial hospital)							
	Community medical service center or township health center	−0.108	0.375	0.084	0.772	0.897	0.431	1.870
	County-level hospital	−0.448	0.370	1.468	0.226	0.639	0.310	1.319
	Municipal hospital	−0.161	0.362	0.197	0.657	0.851	0.419	1.731
	Satisfaction with own medical alliances (Refer = Not satisfied)							
	Satisfied	18.377	0.323	3240.268	*p* < 0.001	95706077.27	50832646.85	180192333.00
	Generally satisfied	16.163	<0.001	-	-	10464364.22	10464364.22	10464364.22
No	Posts (Refer = Administration and logistics)							
	Clinical	1.975	0.543	13.231	*p* < 0.001	7.206	2.486	20.883
	Medical technology	1.040	0.660	2.484	0.115	2.828	0.776	10.302
	Nursing	1.555	0.583	7.116	0.008	4.737	1.511	14.854
	Professional titles (Refer = No title)							
	Senior	−0.137	0.612	0.050	0.822	0.872	0.263	2.891
	Intermediate	0.043	0.573	0.006	0.940	1.044	0.340	3.210
	Junior	−0.026	0.574	0.002	0.964	0.975	0.317	3.000
	Medical institution level (Refer = Provincial hospital)							
	Community medical service center or township health center	−1.734	0.538	10.387	0.001	0.177	0.062	0.507
	County-level hospital	−0.869	0.393	4.886	0.027	0.419	0.194	0.906
	Municipal hospital	−0.929	0.388	5.739	0.017	0.395	0.185	0.845
	Satisfaction with own medical alliances (Refer = Not satisfied)							
	Satisfied	−3.569	0.582	37.647	*p* < 0.001	0.028	0.009	0.088
	Generally satisfied	−1.629	0.523	9.690	0.002	0.196	0.070	0.547
Uncertain	Posts (Refer = Administration and logistics)							
	Clinical	0.416	0.359	1.343	0.246	1.516	0.750	3.063
	Medical technology	−0.297	0.489	0.369	0.543	0.743	0.285	1.938
	Nursing	0.167	0.385	0.188	0.664	1.182	0.556	2.514
	Professional titles (Refer = No title)							
	Senior	−0.843	0.498	2.859	0.091	0.430	0.162	1.144
	Intermediate	−0.303	0.433	0.489	0.484	0.739	0.316	1.726
	Junior	−0.454	0.431	1.113	0.291	0.635	0.273	1.476
	Medical institution level (Refer = Provincial hospital)							
	Community medical service center or township health center	−1.074	0.430	6.220	0.013	0.342	0.147	0.795
	County-level hospital	−0.606	0.364	2.770	0.096	0.546	0.267	1.114
	Municipal hospital	−0.470	0.354	1.768	0.184	0.625	0.312	1.250
	Satisfaction with own medical alliances (Refer = Not satisfied)							
	Satisfied	−0.550	0.745	0.545	0.460	0.577	0.134	2.485
	Generally satisfied	0.426	0.735	0.336	0.562	1.531	0.363	6.468

**Figure 1:**
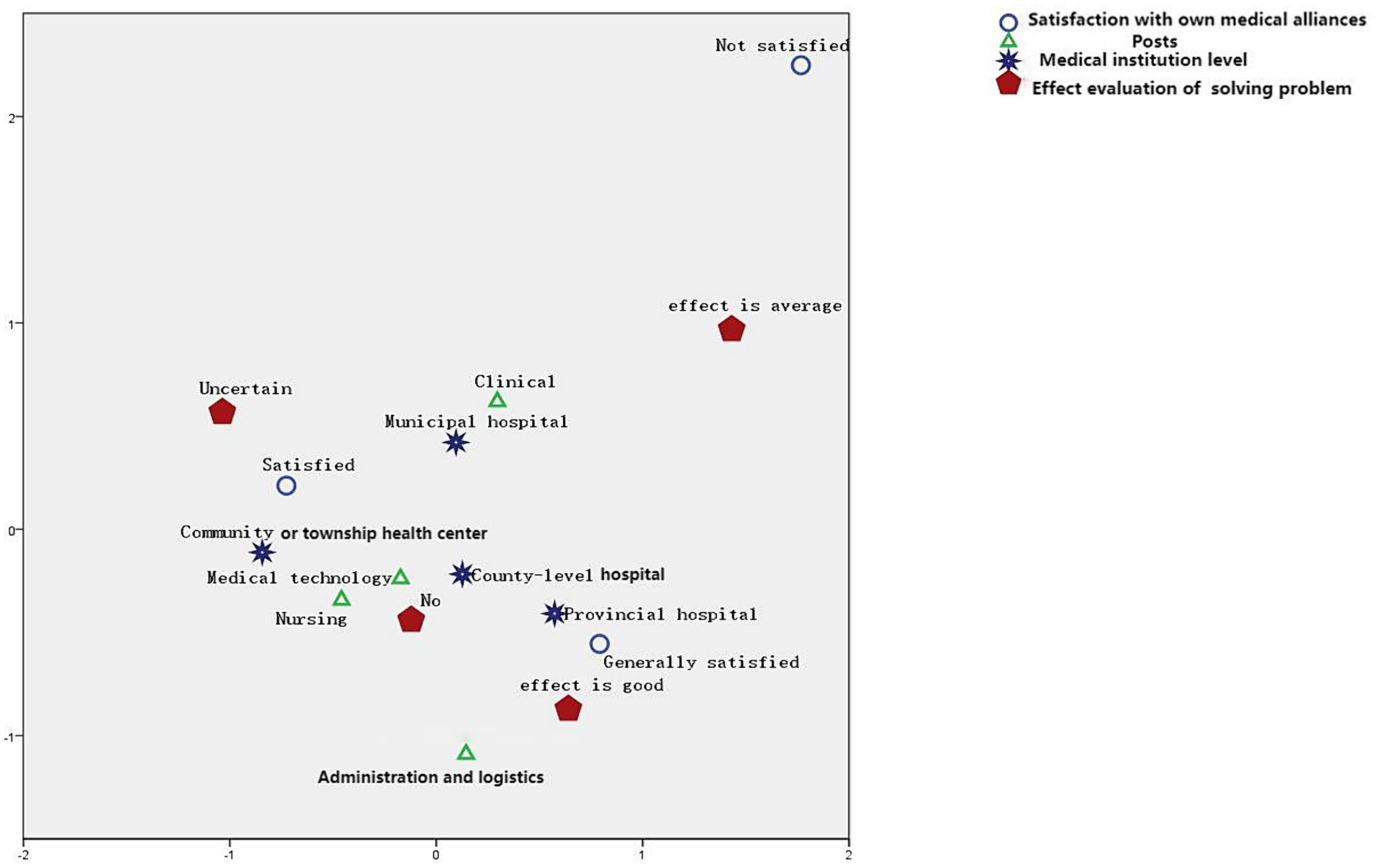
Results of multiple correspondence analysis.

As shown in Figure 1, multiple correspondence analyses showed that medical staff not satisfied with the medical alliances tended to evaluate them as having no effect. Those satisfied with the medical alliances and community medical service or township health centers tended to evaluate the alliances as having a good effect. Moreover, those staffs from county-level hospitals, Medical technology, Nursing tended to evaluate them as having an average effect, and those generally satisfied with the medical alliances tended to be uncertain about the effect.

**Figure 2:**
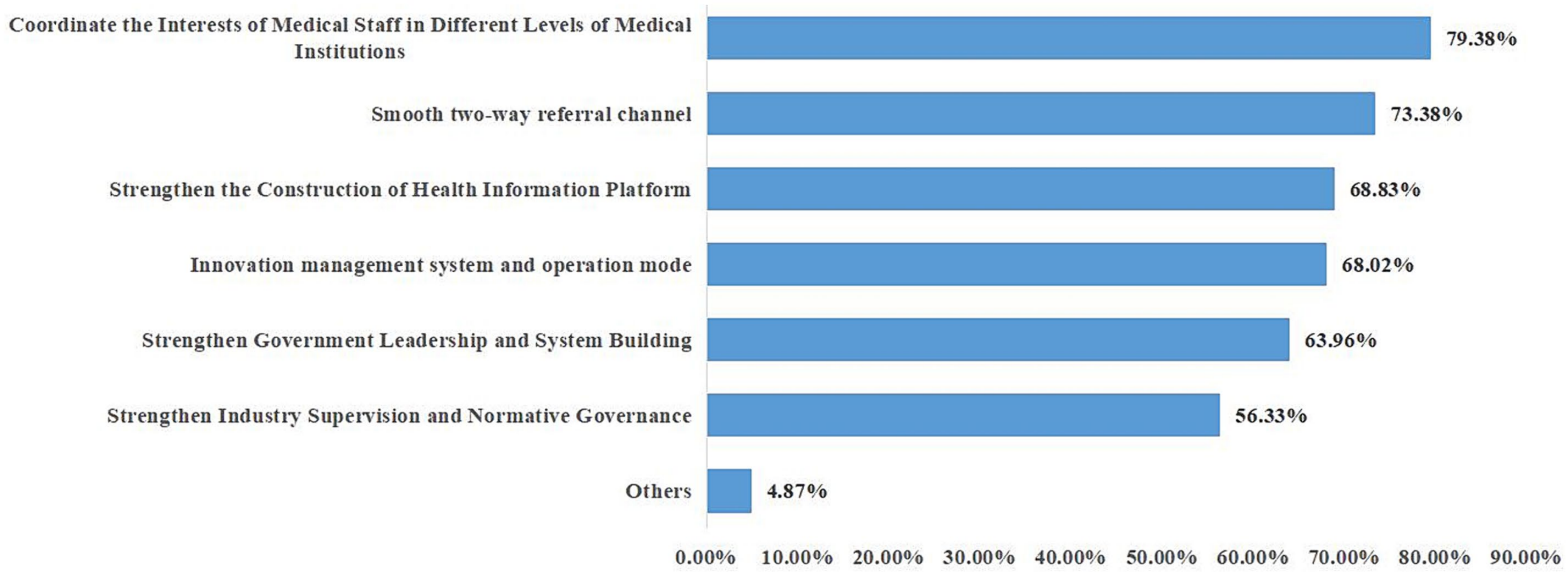
Suggestions of medical staff for the establishment of a medical alliance.

We asked the respondents to suggest further measures to improve the construction of medical alliances. For this, we included relevant multiple-choice questions in the questionnaire. As shown in Figure 2, the medical staff believed that the three most important aspects to improving the formation of medical alliances are the coordination of the interests of the medical staff at different levels of medical institutions, smoothening of two-way referral channels, and strengthening of the construction of health information platforms.

## 4 Discussion

### 4.1 Overall evaluation of medical staff on medical alliances

The study results indicated that 50.00% of the medical staff respondents considered the medical alliances to have an average overall effect on establishing HDT. Only 28.73% of the medical staff believed that the medical alliances could solve the problem of high expenses for medical services and that their effect was good. In addition, 40.10% of medical staff were not satisfied with the medical alliances. Moreover, the medical staff in medical alliances generally believed that the effect of medical alliances on HDTs is average, and they are not very satisfied with the medical alliance. As medical alliances in Fujian Province are still in their initial stages, their effect has not been highlighted. Meanwhile, some studies note a few shortcomings in all aspects of the implementation process of medical alliances, which results in a lack of satisfaction from the medical staff (9). This study highlighted the effect of medical alliances in promoting hierarchical diagnosis and treatment in details from the perspective of medical staff, compared with previous studies. Therefore, it is suggested that more efforts should be made to publicize and train medical staff regarding medical alliances, completely mobilize their enthusiasm for participation, enable them to understand and support the medical care mode of medical alliances, and gradually internalize the medical alliance mode into a sense of empathy and identity (10).

### 4.2 Comparative analysis of different medical staff evaluations of medical alliances in promoting HDT

The study results showed significant differences in the effect evaluation of medical alliances in promoting the establishment of HDTs with different levels of satisfaction (*p* < 0.05). Only 4.12% of medical staff who were satisfied with the medical alliances considered the alliances to have no effect. In contrast, 86.21% of medical staff who were not satisfied with the medical alliances considered the alliances to have no effect.

Furthermore, the study results showed that medical institution level, posts and satisfaction with the medical alliances affected the effect evaluation of medical alliances in solving the problem of high expenses for medical services. Medical staff not satisfied with the medical alliances tended to perceive no effect. Those satisfied with the medical alliances and community medical services or township health centers tended to perceive a good effect. In contrast, those staffs from county-level hospitals, Medical technology, Nursing tended to evaluate them as having an average effect, and those who were generally satisfied with the medical alliances tended to be uncertain about the effect.

Despite the level of awareness about the medical alliance policy or the evaluation of the overall effect of the medical alliance, those in primary hospitals are much more satisfied than those in tertiary hospitals (such as provincial and municipal hospitals), which is consistent with the results of similar studies [i.e., (11–13)]. This may be because the current policy of China’s medical alliance is to adjust and optimize the structural layout of medical resources and promote the sinking of high-quality resources to enhance the service capacity of primary medical institutions and promote HDT (14). Therefore, the formulation of policies and related work is more focused on primary medical institutions, and the interests of primary hospitals are more guaranteed. By contrast, tertiary hospitals must pay for more workforce, materials, and additional financial resources to support primary hospitals. After the hierarchical diagnosis and treatment system is implemented, the economic benefits of tertiary hospitals may be greatly impacted. The workload of medical staff has increased dramatically, leading to more negative performance and lower satisfaction (15). Studies have shown that after the implementation of the HDT, 71.7% of the medical staff believed that the burden of work had increased and that the performance pay did not undergo a reasonable dynamic adjustment (16). The study analyzed different medical staff evaluations of medical alliances in promoting HDT using stakeholders theory from the distribution of benefits, workload changes, medical policies, among others. The results further supplement and refine the existing literature.

### 4.3 Strengthen the construction of medical alliances in various ways

The study results suggest that the three most important aspects of establishing a formal medical alliance are coordination of the interests of the medical staff at different levels of medical institutions, smoothening of two-way referral channels, and strengthening of the construction of health information platforms. Among them, the coordination of the interests of medical staff in medical institutions at all levels is the most essential.

This aspect necessitates the establishment of a restriction mechanism where risks and benefits are shared within the medical alliance. Otherwise, it is difficult to realize the adjustment and integration of resources (17). Furthermore, to improve satisfaction among medical staff, the administration should meet the interests and demands of medical staff and further promote the development of the medical alliance. The interests of medical staff in medical institutions at all levels should be coordinated through risk and benefit sharing. This aims to reduce the impact on the interests of medical staff brought about by the implementation of the medical alliance and help enable the medical staff to cooperate with and support the establishment of the medical alliance mode.

A two-way referral is an important aspect of the work of a medical alliance. Currently, medical alliances lack referral “green channel” services (18), which are critical and form a bridge in implementing two-way referrals. Therefore, to realize two-way referral, it is necessary to establish a smooth and convenient referral channel. A green channel for referral can be established, and special personnel can be appointed to be in charge of arranging and coordinating appointments to clarify the referral principle, indications of patients requiring referrals, and the referral process. Furthermore, quality control indexes of two-way referrals should be perfected to reduce referral resistance and ensure the standardization and effective operation of two-way referrals.

The construction of a health information platform is an essential measure for the development of medical alliances. According to the survey data, the frequency of various activities in medical alliances is uneven, and routine activities such as expert visits and further studies are carried out frequently. Moreover, remote consultation and mutual recognition of examination results require further enhancement (19). Therefore, modern information technology should be adopted to improve the medical alliances’ resource integration and resource sharing. This allows for the broadening of the information cooperation channels between medical alliances and a regional information-sharing platform centered on the regional laboratory center, imaging center, pathology center, residents’ electronic health records, and electronic medical records (20) to realize the effective operation and scientific management of the medical alliances.

Building on existing literature, this study put forward targeted and practical policy recommendations to strengthen the construction of medical alliances in various ways. By doing so, it established the need for policy changes that could positively influence healthcare in society.

The study’s limitation is its small sample size; only 616 medical staff personnel in Fujian Province were interviewed. In addition, the questionnaire content can be further enriched.

## 5 Conclusion

Compared to current research, our study makes a significant contribution to the literature because it highlights the effect of medical alliances in promoting HDT. In summary, medical professionals at primary hospitals evaluated medical alliances’ promotion of HDT much better than those at tertiary hospitals. Given that primary medical institutions are more inclined toward the formulation of policies and related work, the interests of primary hospitals can be guaranteed. Moreover, tertiary hospitals must supply more workforce, material, and financial resources to support primary hospitals. Therefore, it is necessary to coordinate the interests of the medical staff at different levels of medical institutions.

## Data availability statement

The raw data supporting the conclusions of this article will be made available by the authors, without undue reservation.

## Ethics statement

The studies involving humans were approved by all methods were conducted in accordance with relevant guidelines and regulations. Ethical clearance (2022KY137) was granted by the ethics committee of Fujian Medical University Union Hospital. The studies were conducted in accordance with the local legislation and institutional requirements. Written informed consent for participation was not required from the participants or the participants’ legal guardians/next of kin in accordance with the national legislation and institutional requirements.

## Author contributions

ZZ: Conceptualization, Data curation, Investigation, Methodology, Software, Writing – original draft. XX: Conceptualization, Data curation, Investigation, Supervision, Writing – original draft. QW: Methodology, Project administration, Supervision, Writing – original draft, Writing – review & editing.
